# Laparoscopic Cholecystectomy Versus Open Cholecystectomy in Acute Cholecystitis: A Literature Review

**DOI:** 10.7759/cureus.45704

**Published:** 2023-09-21

**Authors:** Raam Mannam, Rajagopal Sankara Narayanan, Arpit Bansal, Vishnu R Yanamaladoddi, Sai Suseel Sarvepalli, Shree Laya Vemula, Saikumar Aramadaka

**Affiliations:** 1 General Surgery, Narayana Medical College, Nellore, IND; 2 Research, Narayana Medical College, Nellore, IND; 3 General Surgery, Narayana Medical College and Hospital, Nellore, IND; 4 Research, Anam Chenchu Subba Reddy (ACSR) Government Medical College, Nellore, IND; 5 Internal Medicine, Narayana Medical College, Nellore, IND

**Keywords:** laparoscopic cholecystectomy, open cholecystectomy, conversion rate, bile leak/injury rate, major complication rate

## Abstract

Cholecystectomy is a common surgical procedure performed worldwide for acute cholecystitis. Acute cholecystitis occurs when the cystic duct is obstructed by a gallstone, which causes gallbladder distension and subsequent inflammation of the gallbladder. Acute cholecystitis is characterized by pain in the right upper quadrant, anorexia, nausea, fever, and vomiting. Cholecystectomy is the treatment of choice for acute cholecystitis. The two commonly performed types of cholecystectomies are open cholecystectomy and laparoscopic cholecystectomy. However, the approach of choice widely fluctuates with regard to various factors such as patient history and surgeon preference. It is imperative to understand the variations in outcomes of different approaches and how best they fit an individual patient when deciding the technique to be undertaken. This article reviews several studies and compares the two techniques in terms of procedure, mortality rate, complication rate, bile leak/injury rate, conversion rate, and bleeding rate.

## Introduction and background

Acute cholecystitis (AC) occurs when the cystic duct is obstructed by a gallstone. This causes gallbladder distension and subsequent chemical or bacterial inflammation of the gallbladder. Gallstones are one of the most common gastrointestinal illnesses, affecting around 10% of the population in the West [1,2]. Gallstones affect more than 80% of individuals who are asymptomatic. In 1-3% of patients with symptomatic gallstones, AC develops [3]. AC is characterized by unrelenting discomfort in the right upper quadrant, anorexia, nausea, vomiting, and fever. Gallstones are present in 95% of patients with AC (calculous cholecystitis), while gallstones are absent in 5% of people with AC (acalculous cholecystitis).

Patients who have symptoms that imply AC should undergo an abdominal ultrasonography to confirm the diagnosis. If the first ultrasound is non-diagnostic or to rule out complications or other diagnoses, further imaging modalities (hepatobiliary iminodiacetic acid or CT scan) may be required. The management of AC comprises two aspects, namely, medical and surgical. Medical management includes bed rest, pain relief, antibiotics, and intravenous fluids. Surgical management includes a procedure called cholecystectomy, i.e., the surgical removal of the gallbladder. Cholecystectomy can be done using an open technique or a laparoscopic technique.

John Stough Bobbs (1809-1870), a Civil War physician from Pennsylvania, is credited with performing the first operation on a human gallbladder. He performed a cholecystostomy in Indianapolis in 1867 [4]. Carl Johann August Langenbuch (1846-1901) conducted the first cholecystectomy in West Berlin’s French district on July 15, 1882 [5].

Open cholecystectomy (OC) is being phased out in favor of laparoscopic cholecystectomy (LC) as a treatment for AC. From 0% in 1987 to 80% in 1992, the proportion of cholecystectomies done laparoscopically has increased [6]. Due to the progress of laparoscopic technology, the growing competence and experience of surgeons, shorter hospital stays, and a shorter period for return to regular activities [7-12], open operations have been replaced by laparoscopic methods.

LC came to rise in France and the United States in the late 1980s, and by the beginning half of the 1990s, 80% of general surgeons in the United States had adopted the equipment and procedures [13]. In the case of AC, LC has a complex role and a few drawbacks such as a high conversion rate to open procedure due to inflammation, edema, and necrosis associated with AC which make the procedure more difficult and may lead to an increased probability of postoperative complications [14-17]. Regardless, in 2006, Tokyo guidelines recommended LC as the first line of treatment for AC [18]. In 2013, a revised edition of Tokyo guidelines for AC was published with a primary focus on delivering the best possible surgical treatment by taking into account the grade of disease severity, timing of the procedure, and type of procedure. AC has been classified into three grades, namely, mild, moderate, and severe, depending on the degree of inflammation of the gallbladder [19,20].

There is debate over the ideal time to undergo surgery. Early surgery versus an initial conservative treatment with antibiotics for complete remission of inflammation, followed by a delayed LC several weeks later are the two main options pursued [21]. The rationale behind delayed surgery is that damaged inflammatory tissue is more sensitive to surgical procedures, which increases the risk of surgical complications. As a result, in the early years of its development, LC was considered contraindicated in AC. LC or OC is conducted according to the patient’s condition, the degree of inflammation, the timing of surgery, and the surgeon’s experience and skill.

This article aims to provide a comprehensive review of the surgical interventions available for the management of AC. Specifically, it intends to compare the efficacy of OC with LC as a therapeutic option for AC and express opinions based on the available published studies.

## Review

Open approach

John Stough Bobbs (1809-1870), a Civil War surgeon from Pennsylvania, is credited with performing the first operation on a human gallbladder. In 1867, he conducted a cholecystostomy in Indianapolis [22]. Carl Johann August Langenbuch (1846-1901), as chief of the French section of West Berlin, conducted the first cholecystectomy on July 15, 1882. Langenbuch had tested the procedure on animals and cadavers before attempting it on humans. Langenbuch submitted a group of 24 patients who had cholecystectomies to the German Surgical Society’s Eighteenth Congress in 1889, claiming that their outcomes were superior to those of other cholelithiasis surgeries at the time. Cholecystectomy, according to Langenbuch, removed both the harmful gallstones and the organ that produces them. “Chirurgie der Leber und Gallenblase” (Surgery of the Liver and Gallbladder) was his debut book, published in 1894 [23].

The ability to evaluate the gallbladder and biliary system before performing surgery is crucial in determining good candidates for cholecystectomy. When Reich [24] inserted bismuth paste and petrolatum into a biliary fistula in 1918, the biliary system was first visualized radiographically. In 1924, a surgical resident working in Evarts Graham’s laboratory, Cole [25] produced the first positive photograph of a human gallbladder.

Following the introduction of LC, numerous relative contraindications emerged, including prior upper abdominal surgery, pregnancy, and cirrhosis. Following this, it has been demonstrated that LC is both viable and secure, if not surpassing, in all of these contexts [7-12]. After 15 years of advancements in technology and increasing expertise with LC, there are few justifications for opting for an open operation. The primary indication for performing an OC is now the conversion from a laparoscopic approach due to failure to complete the procedure laparoscopically. Surgeons opt for the open approach when confronted with inquiries pertaining to anatomy. The indications for conversion encompass a range of factors, such as the presence of severe inflammation, adhesions, anatomical abnormalities, bile duct injury, retained bile duct stones which are difficult to remove laparoscopically, and uncontrolled bleeding.

There are only two specific illnesses connected to the gallbladder that necessitate the implementation of scheduled OC. The suspicion of gallbladder carcinoma is commonly considered a compelling rationale for performing OC [26,27]. If gallbladder cancer is diagnosed intraoperatively, the operation should be converted to an open procedure. Theoretically, an open procedure allows a more controlled performance, with fewer chances of spillage. When there is a suspicion of gallbladder carcinoma based on previous imaging, it is recommended that the patient be referred to a surgeon who is prepared to perform a radical cholecystectomy. This procedure involves a cholecystectomy, resection of segment IV/V of the liver, and lymphadenectomy of the hepatoduodenal ligament [27]. The identification of Mirizzi syndrome, which is the second leading indication for OC, poses challenges in preoperative diagnosis [28]. Laparoscopic procedures have been shown to be helpful in the treatment of type I Mirizzi syndrome with extrinsic hepatic duct compression when performed by skilled practitioners [29,30]. Whether discovered preoperatively or postoperatively, type II Mirizzi syndrome (cholecystobiliary fistula) retains a clear rationale for OC [28,31,32]. The management of type II Mirizzi’s choledochal defect can be challenging, often requiring interventions such as biliary-enteric bypass or, in rare instances, novel therapeutic approaches [33,34]. In individuals with cirrhosis, a condition unrelated to gallbladder problems, OC is occasionally planned.

Procedure

OC can be done in two ways: *anterograde *(beginning the dissection medially in the hepatoduodenal ligament) or *retrograde *(from the fundus downward). The antegrade approach, similar to LC, starts with the dissection of the peritoneum covering the Calot triangle to enable the identification of the cystic artery and duct, culminating in the same *critical view* as LC. The gallbladder is released from the cystic plate in the subserosal plane after the artery and duct are ligated and divided (a cholangiogram is done if required). The advantages of early cystic duct/artery dissection include easier identification in a bloodless field and less blood loss when removing the gallbladder from the liver bed. The retrograde (top-down) technique, in which the dissection begins at the fundus and progresses toward the hepatoduodenal ligament, is another OC approach. Because the cystic duct and artery remain the sole attachments after the gallbladder is separated from the cystic plate, this approach permits the exact identification of the cystic duct and artery. When the gallbladder is in a normal state or exhibits modest inflammation, the antegrade technique can be a straightforward and less invasive method, which is particularly advantageous for teaching young trainees. In cases where the junction between the cystic duct and the common duct cannot be adequately visualized or when there is significant acute inflammation resulting in hardening and thickening of the triangle of Calot or when the gallbladder is excessively tense or fragile such that pulling on the ampulla may lead to gallbladder rupture, it is recommended to perform a retrograde removal of the gallbladder starting from the fundus.

Complications

Table 1 summarizes the impacts of procedure length, mortality rate, major complication rate, bile damage rate, and bleeding rate. Wolf et al. [35] examined 136 instances of OC done for AC from 1997 to 2006 and found a 2.9% death rate, a 4.4% serious complication rate, a 2% bile injury/leak rate, and an average surgery duration of 41.3 minutes. Thompson et al. [36] conducted a similar research with 384 patients who underwent OC for AC and reported a complication rate of 6%. Roslyn et al. [37] identified a death rate of 0.17%, a complication rate of 0.2%, a bile injury/leak rate of 0.018%, and a bleeding rate of 0.004% in their evaluation of 42,474 instances of OC performed between 1988 and 1989. Because of the high sample size, the complication rate was possibly lower than in other studies. Cox et al. [38] conducted a retrospective comparative study among 457 patients who underwent OC from 1985 to 1989 and found a mortality rate of 0.3%, an average length of procedure of 73 minutes, a major complication rate of 3.5%, and a bile injury/leak rate of 0.1-0.2%. Jatzko et al. [11] reported a death rate of 0.007%, a major complication rate of 7.7%, a bile injury/leak rate of 0.02%, a bleeding rate of 0.04%, and an operation duration of 60.6 minutes in comparative research performed between 1991 and 1993 among 700 participants. Smith et al. [39] studied 124 patients from 1990 to 1991 and found an 8% complication rate and an average operation time of 62.7 minutes.

**Table 1:** Complications of open cholecystectomy.

Study	Type of study	Time	Number of cases	Length of procedure	Mortality rate	Major complication rate	Bile injury/leak rate	Bleeding rate
Wolf et al. [35]	Comparative study	1997–2006	136	41.3 minutes	2.9%	4.4%	2%	-
Thompson et al. [36]	Comparative study		384	-	-	6%	0%	-
Roslyn et al. [37]	Population-based study	1988–1989	42,474	-	0.17%	0.2%	0.018%	0.004%
Cox et al. [38]	Retrospective comparative study	1985–1989	457	73 minutes	0.3%	3.5%	0.1–0.2%	-
Jatzko et al. [11]	Comparative study	1991–1993	700	60.6 minutes	0.007%	7.7%	0.02%	0.04%
Smith et al. [39]	Retrospective comparative study	1990–1991	124	62.7 minutes	0	8%	0	0

Laparoscopic approach

It took almost a century for laparoscopy and cholecystectomy to be combined, an unfortunate delay owing to technological restrictions, the lack of a biotechnology interface, and surgical advancement stagnation. The pneumoperitoneum was produced by air insufflation via the scope in early laparoscopes, which had a light source at the distal end. Intra-abdominal thermal damage was a severe concern because of the heat created by these early lights. In 1929, Kalk proposed a second puncture site for pneumoperitoneum formation and described several diagnostic and therapeutic laparoscopic techniques. As a result of his unique and inventive efforts, he was called the Father of Modern Laparoscopic Surgery. The origins of LC may be traced back to the early 1980s when Ko and Airans in Chicago and Cuschieri in Dundee separately tested the procedure on animals [40]. The first human LC was performed in 1987 by Philip Mouret of Lyons, France [41].

The indications for LC are congruent with those for the OC treatment, encompassing symptomatic gallstones, chronic cholecystitis, and pancreatitis. To mitigate or restrict iatrogenic consequences during LC, it is imperative to adhere precisely to the contraindications outlined in Table 2 for surgical procedures. Nevertheless, when faced with substantial intraoperative hemorrhaging, differences in the anatomy of the extrahepatic biliary system and associated arteries, severe intraperitoneal adhesions, and uncontrolled bile drainage, the alternative method of planned LC, an OC becomes necessary.

**Table 2:** Contraindications for laparoscopic cholecystectomy.

Contraindications	
Relative	Absolute
Acute cholecystitis	Inability to tolerate anesthesia
Large gallstones >8 cm in size	Uncontrolled coagulopathy
Anatomical variations of the gallbladder	Severe chronic obstructive pulmonary disease
Extrahepatic ducts	Congestive cardiac failure (ejection fraction <20%)
Intraperitoneal adhesions	
Thick gallbladder walls	
Old age	

Many conditions once felt to be absolute contraindications for LC (e.g., gangrenous gallbladder, empyema of the gallbladder, cholecystoenteric fistulae, obesity, pregnancy, ventriculoperitoneal shunt, previous upper abdominal procedures, and cirrhosis) are no longer considered contraindications but require special care and patient preparation by the surgeon and careful weighing of risk against benefit.

Procedure

The first step involves opening the abdomen and placing the trocar. A periumbilical incision measuring either 12 mm or 5 mm in diameter is made. The determination of whether the incision should be made above or below the umbilicus is contingent upon patient-specific factors. Following insufflation of the belly to a pressure of 15 mmHg, a laparoscope is introduced to facilitate the examination of the abdominal cavity by the physician. The patient is positioned in the reverse Trendelenburg position, with the right side of the bed elevated.

Three further trocars are inserted in the following manner: one trocar with a diameter of 5 mm is placed two to three fingerbreadths below the costal border, aligned parallel to the anterior axillary line. Another 5 mm trocar is inserted two to three fingerbreadths below the costal border, along the plane of the midclavicular line. The gallbladder fundus is grasped and retracted toward the patient’s right shoulder using a locking, atraumatic grasper. To determine the appropriate placement of the final trocar, it is important to possess knowledge of the future positioning of the gallbladder, with specific attention to the cystic forms. The last 12 mm trocar is inserted into the peritoneum to the right of the falciform ligament or into the right side of the falciform ligament, in the epigastrium region, near the midline, at a little angle.

The initial separation of adhesions and dissection of the hepatocystic triangle is performed. During LC, it is frequently observed that adhesions might arise from the duodenum, colon, or omentum. By employing traction and countertraction, it is possible to lower them either easily or with significant force.

The gallbladder infundibulum is securely held by a gentle grasper from the midclavicular port, which then applies force to the patient’s right shoulder, while the fundus is pulled back toward the right shoulder using a locked gentle grasper. The peritoneum within the hepatocystic triangle is separated into anterior and posterior halves using hook electrocautery. The combined utilization of hook electrocautery and blunt dissection is employed to effectively excise the fibrofatty tissue located inside the hepatocystic triangle, both in its anterior and posterior regions. The retraction of the infundibulum of the gallbladder toward the patient’s right side for the anterior dissection and toward the left side for the posterior dissection significantly enhances the efficacy of this dissection.

The gallbladder has now been detached from the lower one-third of the cystic plate. A dissection plane is created by retracting the gallbladder in an anterior and rightward direction, i.e., between the neck of the gallbladder and the liver. The dissection of the plane is extended until the inferior portion of the gallbladder is detached from the cystic plate of the liver. This step is implemented to verify the absence of any leftover structures that may enter the gallbladder from the posterior aspect.

**Figure 1:**
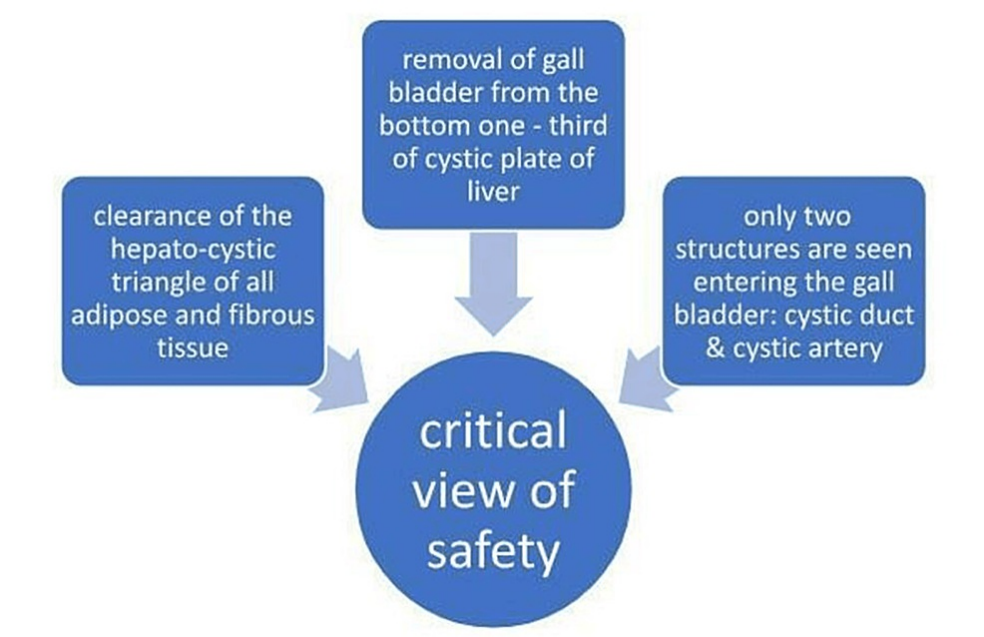
Critical view of safety.

To achieve the critical view of safety (CVS) (Figure 1), a surgeon must be confident that the cystic artery and cystic duct have been correctly identified and are ready to be divided. This includes (1) ensuring that the fibroadipose tissue has been removed from the hepatocystic triangle, (2) the gallbladder has been removed from the lower third of the cystic plate, and (3) only two structures are visible entering the gallbladder-cystic duct and cystic artery [42].

The CVS is not complete if any of these requirements are missing, necessitating further dissection. A bailout treatment, such as an open conversion or partial cholecystectomy, should be seriously considered if the CVS cannot be safely fulfilled, as in the case of significant inflammation [43].

An intraoperative cholangiogram (IOCG) is often done when the biliary anatomy is uncertain, when there is worry regarding choledocholithiasis, and when there is worry about bile duct damage. Whether IOCG avoids bile duct damage is debatable; however, it can identify a bile duct injury intraoperatively [44].

The three criteria for a standard IOCG include (1) In both the right hepatic duct (which includes the right anterior and right posterior sectoral ducts) and the left intrahepatic duct, contrast may be seen above the confluence points of these two vessels; (2) lack of defects in the common bile duct filling; (3) the duodenum receives an unhindered supply of contrast. If any of these are not present, extensive study or action is required.

Following that, the cystic artery and duct are clipped and ligated. After the confirmation of the common bile duct and vascular structures, the subsequent step involves the isolation and separation of the cystic duct and artery. In the conventional approach, it is customary to apply two clips to the residual cystic duct stump, employing a medial-to-lateral orientation. In a similar manner, the cystic artery is secured with two clips on the side opposite the surgical hold, while one clip is applied on the side of the specimen. Subsequently, the use of laparoscopic scissors is employed to divide the structures.

The gallbladder is ultimately removed from the cystic plate, and the port is closed after examining the operating field for bile leak or bleeding.

Typically, the gallbladder is placed into a laparoscopic extraction bag and subsequently extracted. When the gallbladder does not exhibit signs of swelling or inflammation and the presence of relatively tiny stones, prompt removal of the gallbladder by an incision can be undertaken, resulting in reduced susceptibility to wound infection and lower financial burden. Typically, the gallbladder is extracted using a 12 mm periumbilical incision; however, an alternative approach involves removal through a 12 mm subxiphoid port. It is recommended to thoroughly examine the liver bed and clips both before and during gallbladder extraction to verify hemostasis and secure closure of the cystic duct. The port areas can be anesthetized with local anesthesia, after which the ports can be removed while being directly observed.

Complications

Table 3 highlights the implications of conversion rate, mortality rate, major complication rate, and bile damage rate. Smith et al. [39] investigated 1,009 patients from 1989 to 1991 and concluded that the mortality rate of LC was 0.38%, the complication rate was 10.9%, the conversion rate was 3%, and the bile leak/injury rate was 0.5%. Another study performed between 1990 and 1991 among 180 patients by Wilson et al. revealed a death rate of 0%, a complication rate of 9%, a conversion rate of 6%, and a biliary damage rate of 1.1%. The study by Smith et al. indicated a greater complication rate and lower conversion rate than the study by Wilson et al. [46]. Radunovic et al. performed a study from 2005 to 2014 among 740 patients undergoing LC and reported a death rate of 0%, a complication rate of 13.1%, a conversion rate of 3.91%, and a bile damage rate of 1.89%. Agarwal et al. [48] recruited 100 patients from 2017 to 2019 in Udaipur, India, and reported a death rate of 0%, a complication rate of 18%, a conversion rate of 6%, and a biliary damage rate of 6%. Triantafyllidis et al. [49] conducted a study from 2000 to 2008 among 1,009 patients and concluded a death rate of 0%, a complication rate of 9.51%, a conversion rate of 1.39%, and a biliary damage rate of 1.49%.

**Table 3:** Complications of laparoscopic cholecystectomy.

Author of study	Study population	Study period	Number of cases	Mortality rate	Complication rate	Conversion rate	Bile leak/injury rate
Smith et al. [39]	Pennsylvania	1989–1991	1,009	0.38%	10.9%	3%	0.5%
Wilson et al. [46]	Lancaster District	1990–1991	180	0%	9%	6%	1.1%
Radunovic et al. [47]	Montenegro	2005–2014	740	0%	13.1%	3.91%	1.89%
Agarwal et al. [48]	Udaipur	2017–2019	100	0%	18%	6%	6%
Triantafyllidis et al. [49]	-	2000–2008	1,009	0%	9.51%	1.39%	1.49%

Open versus laparoscopic cholecystectomy in acute cholecystitis

There has been continuous controversy for many years over whether LC or OC is the ideal treatment for AC. In the Society of American Gastrointestinal and Endoscopic Surgeons Guidelines released in 1993, AC was deemed a relative contraindication for LC [50]. Since then, as surgical procedures have improved and optical technologies and surgical tools have advanced, LC has been steadily preferred for AC. Tokyo Guidelines 13 (TG13) recommended that LC is better than OC [51].

LC is likely to result in reduced discomfort at incision sites, shorter hospital stays and recuperation times, and improved quality of life compared to open surgery. Regarding costs, laparoscopy is projected to involve higher surgical expenses (cost of disposable equipment) compared to open surgery, but about the same overall costs (direct and indirect medical costs) given the shorter hospital stays and speedier return to society [52]. The choice of surgical method should include surgical risk to the patient, with safety being the first consideration; however, there are several advantages of laparoscopy if the treatment can be performed safely.

Conversion

Only a tiny percentage of OC is done openly; the vast bulk of OC is now accomplished after LC conversion. The range of conversion rates described in the literature is extremely large; nonetheless, in most series, it is less than 10%, and in some closer to 1-2% [53,54]. Several critical risk factors have been reported that predict conversion, including male gender, extreme old age, obesity, prior surgery, severe illness, and emergency LC for AC [54]. Ultimately, however, the individual surgeon is left with their own subjective intraoperative evaluation, evaluating the severity of the inflammatory alterations, clarity of the anatomy, and his or her personal skill and comfort. Despite this, the surgical culture implies that switching to open surgery is a failure. In addition, because of their lack of expertise with OC, many young surgeons may feel more at ease sticking with laparoscopic surgery rather than making the switch. In situations that ultimately need conversion to safely remove the gallbladder, these variables may cause needlessly prolonged operative periods, or worse, unnecessary bile duct injuries.

Converted patients are more likely to experience postoperative infections and other complications, require additional treatments, and have higher readmission rates within 30 days [55]. Determining preoperative patient-related factors and anticipating the need to convert from LC to open surgery can help identify high-risk patients and redefine surgical strategy in this group. Additionally, the conversion from laparoscopic to open surgery results in longer postoperative stays and higher morbidity and mortality rates in this group of patients which is attributed to the underlying cause of conversion [35]. The predicted conversion variables can raise the cost-effectiveness of gallstone therapy and promote patient safety. Standardizing the process of obtaining informed consent and performing preoperative evaluations is crucial for patients with a high probability of undergoing intraoperative conversion. This consistent approach would yield mutual benefits for both the surgeon and the patient.

Some of the conversion variables are age; time of surgery; emergency surgery; comorbid diseases such as diabetes, hypertension, and heart disease; peritoneal adhesions; anatomical variations; and previous abdominal surgery [56]. A statistically important influence is the time of day the procedure is conducted. This is true for surgeries done after 3 p.m. when a complete staff of skilled and knowledgeable surgeons is not present in the hospital ward. Another factor could be the decline in the surgeons’ psychomotor performance, which worsens over the course of a working day, resulting in decreased productivity and less effective surgical outcomes [57,58].

Surgical Difficulty

Severe inflammation of the gallbladder and its periphery enhances both the difficulty of LC and the incidence of postoperative complications. The estimated incidence of significant complications such as bile duct injury (BDI) and vasculobiliary injury is two to five times greater with LC than for OC [52,59]. As AC is a prevalent disease, even if the incidence of complications is modest, the absolute number of patients is significant. Surgical difficulties must be accurately assessed and treatment methods must be standardized if these significant consequences are to be minimized. Many prior studies have employed criteria such as the open conversion rate, operating duration, and the frequency of complications as markers of surgical complexity.

In situations involving anticipated surgical complexity, it is recommended to use a multidisciplinary strategy by engaging several specialists such as radiologists, gastroenterologists, hepatologists, and others. This collaborative effort aims to thoroughly evaluate the patient’s condition before the surgery and minimize the probability of complications.

An evaluation of preoperative data and diagnostic imaging utilizing operating time and the open conversion rate as markers of surgical difficulties in patients of symptomatic cholelithiasis (including AC), preoperative cholangiography revealed the following five parameters that substantially increased the time needed for cholecystectomy: body mass index, non-visualized gallbladder, cystic duct length, temperature, and aberrant CT results [60].

Another study found that impacted stones in the gallbladder neck, thickness of the gallbladder wall, and persistently elevated C-reactive protein levels led to a prolonged operating time. Several further studies have reported that variables such as male gender, elevated white blood cell count, decreased albumin levels, increased bilirubin levels, presence of fluid accumulation around the gallbladder, and the presence of diabetes are indicative of the likelihood of open conversion [61-64].

Several studies have indicated that performing surgery within 72 hours of the initiation of AC is associated with a reduction in complications, shorter operating time, and increased ease of the surgical procedure [65,66]. This is because these studies examined the timing of surgical interventions.

TG07 recommended that surgical intervention for AC should be promptly performed upon hospital admission. However, TG13 recommended that surgery should be undertaken shortly after admission, ideally within 72 hours from symptom onset. When considering the emergence of AC, accurately determining its initial occurrence poses a challenge. Certain patients may only manifest symptoms after 72 hours have elapsed after the first onset.

A significant number of surgeons continue to choose the approach of managing patients with AC by conservative treatment first, followed by delayed cholecystectomy [67]. Notwithstanding this, a notable proportion of patients, specifically 15%, require emergency surgical intervention, while an additional 25% undergo readmission before undergoing elective surgery. According to a study conducted by Cameron et al. [67], approximately one-third of patients undergo readmission during the initial three weeks following their release from the hospital. The findings presented suggest a notable prevalence of issues experienced by those awaiting postponed cholecystectomy. To reduce both the duration and financial burden associated with hospitalization, the implementation of an early cholecystectomy procedure has been suggested as a potentially advantageous approach [68,69].

Limitations

The limitations of this study are that we used only PubMed and Medspace as our database, and the article discusses only a generalized overview of the outcomes described above. It does not acknowledge the specific factors as to why those outcomes occur. The utilization of solely PubMed and Medscape for conducting a review may present inherent biases that could limit the comprehensiveness of the overall analysis. To address this concern, future studies should consider including additional databases to ensure a more comprehensive assessment.

## Conclusions

It is recommended to employ early LC as a potential treatment option for mild AC. The option of performing cholecystectomy either early or with a delay may be taken into account for mild AC. However, it is important to note that early LC should only be performed by surgeons who possess a high level of competence, and if the surgical conditions make it challenging to identify the anatomical structures, it should be promptly converted to OC. For patients diagnosed with severe AC, initial management often involves conservative treatment utilizing antibiotics. In cases where inflammation is confined to a certain extent, it is recommended to proceed with open surgery. Because each patient presents differently and the factors that determine the type of surgical procedure required fluctuate, it is optimal to treat the patient with individualized patient-centered care to provide the best possible care.

Nowadays, almost every conventional technique is shifting toward a modern, minimally invasive technique and cholecystectomy is one among them. In this article, we have cited several studies to describe the indications, techniques, and outcomes, including the complication rate, mortality rate, bile leakage/injury rate, and bleeding rate, among others. This article provides a comprehensive overview of cholecystectomy and assists surgeons in selecting a surgical method when a patient presents with AC. Compared to open surgery, LC is anticipated to result in less pain at incision sites, shorter hospital stays and recovery periods, and enhanced quality of life. Laparoscopy is anticipated to incur greater surgical expenditures (cost of disposable equipment) than open surgery, but similar total costs (direct and indirect medical costs) due to shorter hospital stays and quicker return to society. OC is chosen only when the bladder is highly inflamed and laparoscopic surgery is impossible. Recent trends indicate that the majority of OC procedures are performed through LC conversions and that medical students do not get an adequate education in OC owing to the dominance of LC. In conclusion, both approaches have their own advantages and disadvantages, and a surgeon must choose the technique depending on their expertise and the patient’s desired outcome. The clinical implication of this research is to understand how these varied outcomes vary regarding the type of approach and how a surgeon may tailor them depending on his or her experience and patient profile. As several other variables also influence these results, we urge that further research be conducted in the future to comprehend these disparities among the procedures.
